# Coordination costs may counteract the positive effects of a larger group size in determining the winner of between-group conflicts

**DOI:** 10.1016/j.isci.2026.115496

**Published:** 2026-03-30

**Authors:** Miguel Gareta García, Erica van de Waal, Xiang-Yi Li Richter, Redouan Bshary

**Affiliations:** 1 Department of Ecology and Evolution, University of Lausanne, 1015 Lausanne, Switzerland; 2 Institute of Biology, University of Neuchâtel, 2000 Neuchâtel, Switzerland; 3 Inkawu Vervet Project, Mawana Game Reserve, KwaZulu-Natal 3115, South Africa; 4 Department of Human Evolutionary Biology, Harvard University, Cambridge, MA 02138, USA; 5 Center for Functional Biodiversity, School of Life Sciences, University of KwaZulu-Natal, Pietermaritzburg, KwaZulu-Natal, South Africa; 6 The Sense Innovation and Research Center, Lausanne and Sion, Switzerland; 7 Institute of Ecology and Evolution, University of Bern, 3012 Bern, Switzerland; 8 Department of Biology, University of Konstanz, 78464 Konstanz, Germany

**Keywords:** social interaction, zoology, psychology

## Abstract

Collective defense of territories and resources is a key benefit of group living and is often modeled as a public goods game. Although larger animal groups have the potential to mobilize greater fighting power in between-group competition, this advantage may be constrained by coordination. Our review of quantitative studies shows that, independent of a population’s average group size, larger groups usually win, with few exceptions. We examined one such case in vervet monkeys. As expected, group size was positively correlated with group spatial spread, and more dispersed groups were less likely to win encounters, implicating coordination problems. We then developed a mathematical model showing that, as coordination costs rise with group size due to spatial spread, they can cancel the usual “strength in numbers” advantage of larger groups in between-group conflicts. Our work highlights how between-group competition provides a natural setting for integrating ecological constraints into models of N-player cooperation.

## Highlights

• We combine literature, wild vervet field data, and a theoretical model on group size and between-group contest (BGC) outcomes
• Across primate data, larger groups are generally more likely to win BGC
• Vervet monkeys provide an exception, linked to large groups being spatially spread and less coordinated
• Our model suggests that coordination costs can make smaller groups more successful in BGCs

## Graphical abstract

**Figure:**
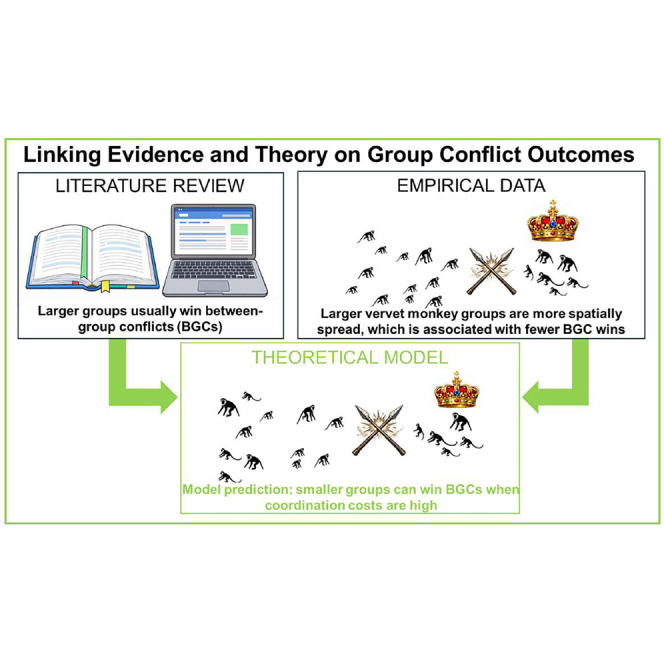

## Introduction

Group living is a prime example of cooperation in a great variety of species. It may have evolved as an anti-predation strategy,^1^^,^^2^ as it can offer benefits such as risk dilution, early predator detection, confusion effects during escape, and cooperative defense.^3^^,^^4^^,^^5^ Additionally, group living can provide foraging advantages, including facilitating cooperative hunting,^6^^,^^7^ sharing information about patchy food resources,^8^ and jointly defending resources against neighboring groups or interspecific competitors,^9^^,^^10^ making within-group social dynamics a field of great conceptual interest. While most empirical and theoretical research focuses on interactions between pairs of individuals,^11^^,^^12^ group-level interactions involve multiple individuals. Therefore, an individual’s action may affect the payoffs of several other individuals, a situation we refer to as an N-player game. This leads to a primary challenge for helpful individuals: avoiding exploitation by partners who do not reciprocate.^13^^,^^14^ Such uncooperative individuals are often referred to as defectors, cheaters, or free riders.

Theoretical research on payoff structures and strategies in N-player games offers three broad categories: N-player prisoner’s dilemmas (NPDs), non-linear public goods games (NLPGGs) such as the volunteer’s dilemma, and by-product benefit games. In a standard NPD game, each individual in a group decides how much, if anything, to contribute to a shared pool in each round. Contributions generate linear added value, which is then equally distributed among all group members, regardless of individual contribution variation. When an individual’s contribution costs exceed the added value divided by the number of group members, this game offers a vast parameter space in which helping is a biologically altruistic act,^15^^,^^16^ reducing the helper’s lifetime direct fitness. Potential cooperative solutions to NPDs involve combining the game with additional interactions, enabling players to punish or gain reputation,^17^^,^^18^ or making assumptions about population structure that result in between-group competition and/or a genetic structure in which individuals can increase their inclusive fitness by contributing to the public good.^19^ In these cases, distinguishing between direct and indirect fitness benefits poses a significant challenge for empiricists.^20^

For instance, while NPD models assume a linear relationship between contributions and created value, the relationship is often sigmoidal.^21^^,^^22^ Under such conditions, the resulting payoff matrix aligns with an NLPGG in which contributing to the public good is subject to negative frequency-dependent selection.^23^ A simple but extreme example of an NLPGG is the volunteer’s dilemma, in which a single cooperator is necessary and sufficient to produce the public good.^23^ Predator scanning is a classical case, as only a few vigilant individuals are required to ensure group-wide safety, allowing others to continue foraging.^24^^,^^25^

Another factor shaping the payoffs of cooperation is the unequal distribution of benefits among group members, often driven by spatial structure. For example, in meerkats (*Suricata suricatta*), individuals located nearest to the sentinel obtain the largest benefits from early predator detection, while those further away receive delayed or reduced information.^26^^,^^27^ Similar spatial heterogeneity is observed in primates, where alarm calls degrade with distance or vegetation density, providing the greatest anti-predator advantage to nearby individuals.^28^^,^^29^^,^^30^ Cooperative hunting in bottlenose dolphins (*Tursiops truncatus*) shows the same principle, where individuals occupying central positions in the prey-herding formation gain greater access to tightly clustered fish, whereas peripheral individuals benefit less from the collective effort.^31^ These cases illustrate that even when cooperative acts generate a group-level public good, their benefits may remain spatially localized, producing uneven payoffs across individuals. Similar patterns are seen in microbial systems, where bacteria releasing extracellular enzymes disproportionately benefit their nearby neighbors, making the behavior effectively self-serving rather than altruistic.^32^^,^^33^

In contrast, the role of group spatial spread distribution (also known as spatial cohesiveness) in shaping cooperation is less well understood, even though it can fluctuate with seasonal conditions, food availability, and activity patterns such as travel, feeding, and socializing.^34^^,^^35^^,^^36^^,^^37^^,^^38^^,^^39^ Group spatial spread has implications for individuals’ feeding ecology and risk of predation (i.e., herd effect).^40^ Hence, a group’s resulting spatial distribution depends on trade-offs that reflect the challenge of satisfying divergent individual needs within the group.^41^^,^^42^^,^^43^ These trade-offs generate conflicts of interest over where to position oneself and how to move with respect to others, naturally giving rise to collective action problem-like situations that warrant cooperative solutions.^44^^,^^45^^,^^46^^,^^47^

Between-group conflicts (BGCs henceforth) provide a valuable example for studying within-group N-player cooperation games, both empirically and theoretically. Indeed, empirical data have helped refine the often-stylized models by adding key parameters to explore how varying their values affects social behavior. There is an extensive body of literature in microeconomics that explores conflicts among human groups, such as sports teams, competing firms, and political groups,^48^^,^^49^^,^^50^ complemented by models aimed at understanding BGCs in non-human animals.^51^^,^^52^ A critical insight from such integrative efforts is that larger groups tend to win conflicts when benefits are shared according to relative contributions. Importantly, variation in contributions may result from a within-group hierarchy that regulates access to resources, leading high-ranking individuals to contribute more because they have more to gain from winning. Hierarchies are thus one factor that impacts the problem of free riding, which is frequently emphasized in the NPD literature.

BGCs occur in various animal taxa.^53^^,^^54^ Studies typically distinguish between territorial conflicts—over an exclusively used area^55^^,^^56^^,^^57^^,^^58^—and resource conflicts, such as the defense of a fruiting tree located in the overlap zone of two home ranges.^59^^,^^60^^,^^61^^,^^62^ BGCs can involve aggression and occasionally be life threatening in some animal groups, including central chimpanzees (*Pan troglodytes troglodytes*),^63^ banded mongooses (*Mungos mungo*),^64^ meerkats,^65^ or crested macaques (*Macaca nigra*).^66^ A substantial body of empirical literature examines the relationship between relative group size and the likelihood of winning a BGC, thus gaining access to resources and therefore acquiring more net energy, leading to increased reproductive success.^54^^,^^67^ A qualitative analysis in primates supports the intuitive prediction that groups that are larger groups than their opponents are more likely to win due to their greater number of individuals available for participation in BGCs.^54^ However, the benefits of winning BGCs, such as gaining access to a contested food resource, are often considered a public good, raising questions about individual contributions and how the gains are shared.

The factors influencing individuals’ motivation to participate in BGCs can vary according to several characteristics, including sex,^68^^,^^69^^,^^70^^,^^71^^,^^72^ age,^68^^,^^73^ dominance rank,^71^^,^^74^^,^^75^^,^^76^ reproductive state,^69^^,^^77^ having dependent infants,^69^^,^^75^^,^^78^ or the presence of close social bonds.^79^^,^^80^ Participation may also be affected by external factors such as food abundance^69^^,^^72^^,^^78^ or the risks involved.^79^^,^^81^^,^^82^ In the species we investigate in some detail, vervet monkeys (*Chlorocebus pygerythrus*), groups are more likely to defend areas with abundant food resources,^83^ and this behavior is observed among both male and female individuals.^78^ Strict, linear dominance hierarchies characterize vervet monkeys,^84^^,^^85^ and females’ dominance increases their participation in BGCs,^78^ although the link between male rank and contributions has not yet been investigated.

Consequently, BGCs offer an excellent opportunity to examine how the state of key variables affects payoff distributions among group members and, subsequently, influences optimal individual strategies regarding participation and the probability of the larger group’s success. Our study comprises three components. First, we provide some evidence from quantitative empirical data published on BGCs in group-living animals that include diverse species such as gray wolves (*Canis lupus*), Ethiopian wolves (*Canis simensis*), spotted hyenas (*Crocuta crocuta*), meerkats, lions (*Panthera leo*), capuchin monkeys (*Sapajus nigritus*), or vervet monkeys, focusing on whether the specific publications present analyses within each system report that numerically larger groups tend to win more often (Table S1). As shown in the following paragraph, these study-level summaries generally confirm the qualitative evaluation of primate species that larger groups are more likely to win,^54^ independently of the mean group size of the studied population. However, there are few exceptions, most prominent in vervet monkeys.^83^

We conducted detailed analyses of a vervet monkey population at the INKAWU Vervet Project (IVP) in South Africa to identify factors that might explain why BGC patterns in this species depart from those observed in other taxa. These analyses focus on data from wild groups and are structured into two main sections. First, we examine whether larger groups (total group size) occupy more extensive areas (see larger groups have a larger group spatial spread section), where we expect that larger groups will cover larger areas. Second, we assess how group spatial spread, food abundance, and seasonality affect the likelihood of winning BGCs (see groups that are less spread are more likely to win section), predicting that greater spatial spread hampers group coordination during BGCs and reduces the chance of victory (see hypotheses and predictions in Table S2).

Our vervet monkey case study suggests that theoretical research on N-player cooperation may have overemphasized cheating while overlooking the challenges of coordination in larger groups. We then develop a theoretical model that investigates how group size-dependent coordination costs due to limited patch sizes and the division of resources among participants shape which groups are more likely to win. Although resource distribution can, in principle, follow many forms (e.g., equal sharing, rank-based allocation, or contribution-based rewards), our model focuses on the egalitarian case, in which all group members receive the same payoff. Under this assumption and given that coordination costs rise with total group size if limited patch size causes increased group spatial spread, individuals join a BGC only when their expected net gain is positive. This mechanism determines the realized fighting-group size and thereby the probability of victory. The model shows that larger groups tend to win across a vast parameter space but also identifies conditions under which smaller groups can prevail.

## Results

### Literature review

For cross-species comparison, we recorded the mean total group size reported for each study/population and the authors’ quantitative conclusion on whether numerically larger groups tended to win more often. Because detailed, contest-by-contest relative group sizes were seldom available, we did not attempt to model winning probability as a function of group size; instead, each study contributes a single binary data point (“larger group wins more often” versus “not”). We identified 33 studies that reported mean total group size of the study population, contest type, and quantitative outcomes of BGCs. Of these, 13 examined resource contests (39.4%), while 20 focused on territorial contests (60.6%).

**Figure 1:**
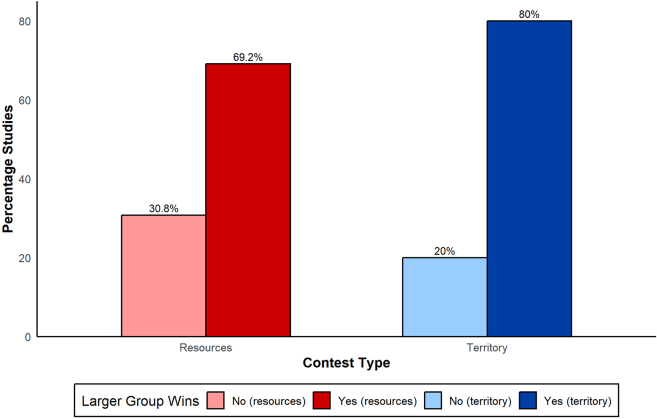
Larger-group advantage across contest types The proportion of studies reporting that larger groups win more often (y-axis) is shown separately for resource and territorial contests (x-axis). Most studies found a larger-group advantage, particularly in territorial contests compared to resource contests. Bars represent the percentage of studies (N = 33; Table S1) reporting that larger groups won more often (“Yes”, dark colours) or not (“No”, light colours). Colours indicate contest type (resource = red; territorial = blue). Each bar summarises binary study-level outcomes (larger group wins: yes/no), rather than contest-level probabilities.

Across all studies and species, most found that larger groups won more often than expected by chance (25/33 studies, 75.8%), while the remainder found no such effect (8/33, 24.2%). This trend was evident across both contest types, and was more pronounced in territorial contests (80% of studies) than in resource contests (69%; Figure 1). Among the eight studies in which larger groups did not win significantly more frequently, seven reported no detectable effect of group size on the odds of winning. Only one study—on vervet monkeys—found that the smaller group won more frequently; importantly, this pattern was based on within-contest size differences rather than on overall mean group size at the population level.^83^

These findings provide only a coarse, study-level summary: each study contributes a single binary data point indicating whether, across the encounters analyzed, larger groups were significantly more likely to win. Nonetheless, the data are consistent with the general idea that larger groups tend to win more often, particularly in territorial contests, while also showing that this pattern is not universal. Next, we investigate which underlying factors might explain why the general tendency for larger groups to win BGCs did not hold in a minority of the studies we reviewed and was even reversed in vervet monkeys.^83^ In particular, we asked which additional parameters could offset or reverse the positive effect of total group size under certain conditions, and we explore the role of such parameters in the study in which smaller vervet monkey groups won BGCs more frequently.

### Case study of BGCs in vervet monkeys

#### Larger groups have a larger group spatial spread

**Figure 2:**
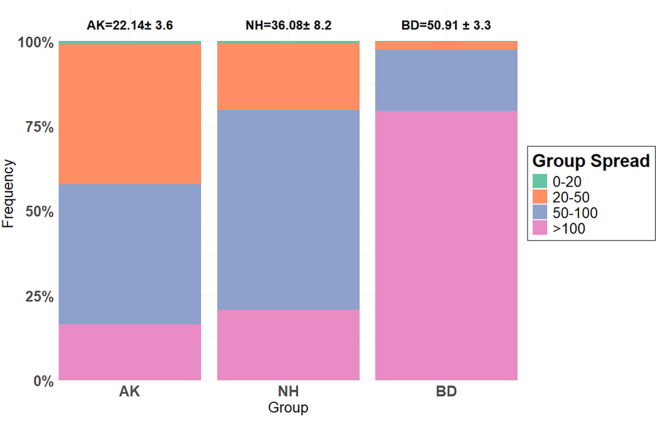
Group spatial spread across vervet monkey groups of different sizes The distribution of group spatial spread (y-axis) is shown for three vervet monkey groups (x-axis) of increasing total size: AK, NH, and BD. Larger groups tended to exhibit greater spatial spread, with a higher proportion of observations in wider distance categories. Bars represent the proportion of observations within each spatial spread category (0–20 m, 20–50 m, 50–100 m, >100 m), indicated by green, orange, blue, and pink, respectively. Mean ± S.D. group size for each group over the study period is shown above each bar (related to Figure S5).

We analyzed data on three groups of wild vervet monkeys (AK = Ankhase, NH = Noha o, and BD = Baie Dankie) from the IVP in the Mawana Game Reserve, South Africa. The cumulative link mixed model revealed a strong positive association between total group size and group spatial spread, with this association differing across seasons. In summer, larger groups were more likely to be recorded in higher spatial spread categories (slope for group size = 0.15 ± 0.01, z = 19.3, *p* < 0.001). In winter, this effect was even stronger (slope for group size in winter ≈0.20, obtained as the sum of the group-size main effect and the interaction term; interaction term = 0.04 ± 0.007, z = 5.78, *p* < 0.001), whereas the intercept shift between seasons was negligible (season effect = −0.03 ± 0.32, *p* = 0.93). Thus, larger groups were more spatially spread than smaller groups in both seasons, with a steeper size-spatial spread relationship in winter. Model predictions further show that small spatial spreads (0–50 m) are increasingly rare, while very large spreads (>100 m) become more common as group size increases, especially in winter (Figure S1). Consistent with these model results, the distribution of group spatial spread categories differed across groups of increasing total size: distances of 20–50 m were most frequent in the smallest group (AK), spreads of 50–100 m dominated in the intermediate group (NH), and spatial spreads greater than 100 m were most common in the largest group (BD; Figure 2).

#### Groups that are less spread are more likely to win

**Figure 3:**
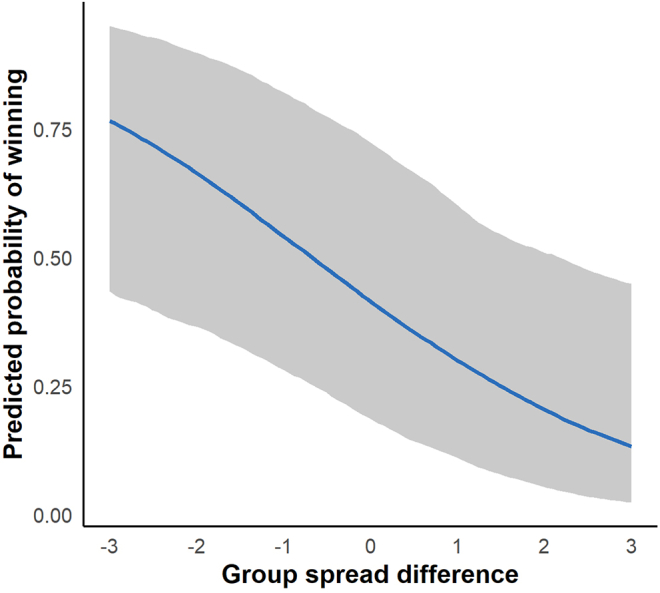
Group spatial spread differences predict between-group contest outcomes The predicted probability that the focal group wins a between-group contest (y-axis) is shown as a function of the difference in spatial spread between the focal and rival groups (x-axis). Negative values indicate that the focal group is less spread out than its rival, whereas positive values indicate the opposite. Focal groups were more likely to win when they were less spread out than their opponents. The x-axis represents group spread difference (Δspatial spread = focal category − rival category; range −3 to +3), based on four vervet groups (AK, NH, BD, CR). Spatial spread categories are defined as <20 m (1), 20–50 m (2), 50–100 m (3), and >100 m (4). The solid line shows posterior mean estimates from the Bayesian model, and the shaded area indicates the 95% credible interval (related to Table S4; Figures S2 and S3).

Using data from four vervet groups (AK, NH, BD, and CR; see STAR Methods), the Bayesian additive model showed that a more negative group spatial spread difference of the focal group relative to the rival (that is, the focal group being less spatially spread out than its opponent) was strongly associated with an increased probability of winning a between-group contest (posterior mean = −0.52, 95% credible interval: −0.94 to −0.12). This indicates that as focal groups become more spatially cohesive or less spatially spread compared to their rivals, their odds of winning a BGC increase (Figure 3). By contrast, the effects of season and relative NDVI (normalized difference vegetation index, a satellite-derived proxy for local vegetation greenness and food availability) at the contest location showed no clear support in the data (95% credible intervals included zero).

### Modeling reveals conditions where coordination benefits small groups in BGCs

**Figure 4:**
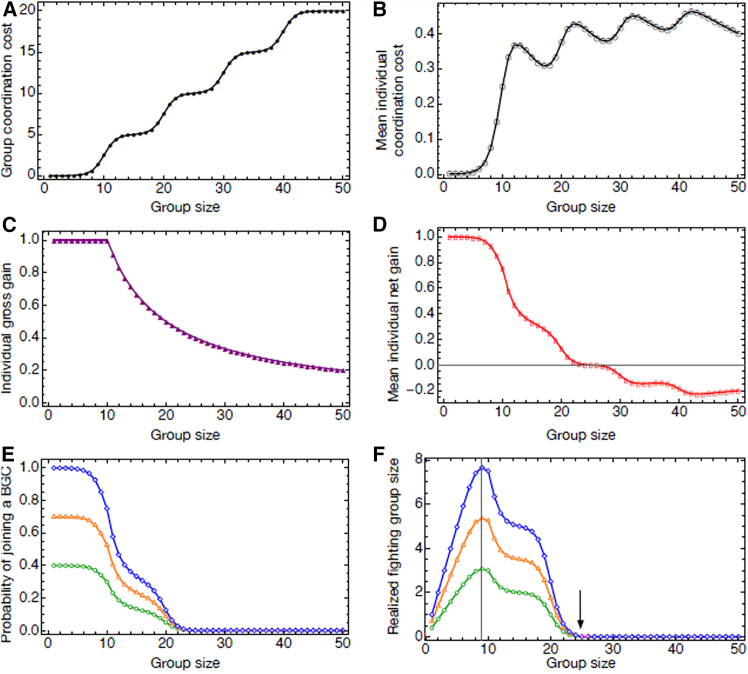
Model predictions linking group size, coordination costs, and contest participation Ilustrations of the mathematical model showing how coordination costs, individual gains, and participation in between-group contests vary with group size. (A) Coordination costs increase with group size in a stepwise manner when all members participate. (B) Average individual coordination cost varies non-linearly with group size. (C) Expected individual gross gain from a successful contest assuming equal resource access. (D) Expected individual net gain decreases with group size. (E) The probability of joining a contest increases with positive net gain and drops to zero when coordination costs exceed expected gains. (F) Realized fighting group size initially increases and then declines once total group size exceeds a critical threshold. Lines show model predictions for different values of α (0.4, 0.7, 1.0), with other parameters held constant (C = 5, k = 1, n₁ = 10, n₂ = 20, n₃ = 30, n₄ = 40, v = 10). Parameter definitions are provided in the STAR Methods.

To complement the vervet analyses (see “larger groups have a larger group spatial spread” and “groups that are less spread are more likely to win”), we developed a simple mathematical model to explore whether smaller groups can have an advantage in BGCs because larger groups face larger coordination costs. The model introduces food patches (*v*) where the size indicates the resources available in each local cluster (e.g., the number of seedpods or fruits on a tree). As long as group sizes are below the carrying capacity of food patches, the group can stay cohesive and move together from one patch to the next. In contrast, if group size exceeds the carrying capacity of food patches, group members need to spread out over two or more patches. This spread causes coordination costs when a group should aggregate at a resource contested by another group. In species that can live in groups much larger than the maximum number that can feed at the average food patch, the distribution of individuals can spatially spread more, leading to an increase in coordination cost following a “staircase-like” pattern (Figure 4A), assuming all individuals would join a fight. Here, each step corresponds to a cluster of individuals in a food patch (i.e., a tree) within the group’s territory. The model assumes that the more patches are occupied, the more the group is spread, and hence the higher the costs of joining for individuals furthest away. Consequently, the average individual cost of coordination is also a non-linear function of group size, and we assume that this cost is slightly reduced as more individuals from a single cluster join the fight (Figure 4B). Importantly, groups larger than the patch’s carrying capacity not only incur coordination costs but also face limited resource value from a BGC when the accessible patch cannot provide enough food for all group members. Here we assume that, following a won BGC against a rival group, all members of the winning group share this resource regardless of whether they contributed to fighting; the expected gross individual gain decreases with group size (Figure 4C). The flat part of the curve reflects the maximum resources an individual must consume to reach satiation. Subtracting the mean coordination cost from the expected gain yields an individual’s mean expected net gain (Figure 4D).

The expected net gain will influence an individual’s motivation to participate in a BGC. Therefore, we model the mean probability of joining a fight as proportional to the expected individual gain, with a scaling factor that adjusts the overall level of individual willingness to participate, which can be a species characteristic (Figure 4E). Multiplying this probability by group size yields the expected fighting group size, which first increases and then generally decreases as the total group size exceeds the carrying capacity of the food patch (Figure 4F). We assume that a contesting group with more individuals participating in fighting is more likely to win because of the increased fighting power and the reduced risk of injury (the latter may lead to the smaller fighting group retreating without overt aggression). We find that if the contested resource offers enough food for all group members to reach satiation (Figure 4F, vertical gray line), larger groups tend to win because all individuals are likely to join the fighting. However, there is a critical patch size at which average gains per group member begin to drop. Because of the resulting reduction in mean motivation to join and the resulting lower fighting group size above this threshold, smaller groups tend to win under such circumstances. If one group is smaller than the critical patch size can supply, while the opponent group is larger, group size alone is insufficient to predict which group is more likely to win. Finally, since the coordination cost will eventually exceed the value of the contested resource as group size increases, there is an upper limit to group size at which BGCs can occur, as illustrated by the black arrow in Figure 4F. Note that this conclusion is tightly linked to the assumption that all group members can share the resource in case of victory; if dominants can monopolize the resources or if non-participants do not join in the case of victory, no such upper limit would be predicted. These results should be interpreted at the level of a single ecological context with a given patch size *v*: the vertical gray line in Figure 4F marks the group size at which the capacity of a typical food patch is reached. Species can differ both in their typical group sizes and in the size and distribution of food patches, so our model does not predict a simple monotonic relationship between species’ mean group size and the probability that larger groups win across taxa.

## Discussion

Our study examined how well total group size predicts the likelihood of winning BGCs in non-human animals and whether additional factors improve these predictions. Most quantitative studies show a positive relationship between mean group size and winning probability. However, having more members than a rival is not an infallible predictor: several studies report no effect, and one even found higher success rates for smaller groups.^77^^,^^83^ We explored this one study in more detail and identified increased group spread in larger groups as a potential factor favoring smaller, spatially more cohesive groups. A model that puts group spread in relation to group size interacting with patch size indeed identifies conditions under which relatively smaller groups may be favored.

### Insights from the literature review

Group-living animals typically engage in two forms of defense: territorial and resource defense.^62^^,^^76^^,^^86^^,^^87^ Territorial defense is favored when economically feasible and shaped by individual investment, group size, and home-range characteristics, with species varying widely in how defensible their territories are.^10^^,^^58^^,^^62^ Resource defense, by contrast, is more likely when monopolizable or patchy foods make defense profitable.^62^^,^^88^^,^^89^ Both defense types can involve public goods and thus rely on collective action.^10^^,^^45^^,^^76^^,^^77^^,^^90^^,^^91^ When group members differ in their incentives to participate, collective-action problems can emerge, leading to unequal contributions and, in some cases, individuals opting out of defense.^10^^,^^45^^,^^76^^,^^77^^,^^90^^,^^91^

Territorial losses can impose long-term costs on all group members, promoting widespread participation and reducing free riding. In contrast, losing access to a current resource has no lasting consequences and often affects individuals unevenly,^51^ so group size is expected to play a weaker role in resource defense. In our limited dataset, studies on species with larger typical group sizes were only slightly more likely to report a larger group advantage in territorial contests than in resource contests (Figure 1), and this trend was weak. Broader comparative work across more species and a wider range of group sizes is needed to clarify how contest type and relative group size shape potential trade-offs. Although larger groups (relative to their rivals) tend to win more often, this pattern—based on just 33 studies—does not depend solely on average group size. Two factors may help explain why larger groups do not always prevail. Some contested resources benefit only a subset of individuals, and larger groups may incur higher coordination costs when synchronizing collective responses.

### Why groups that are larger than their rivals often win

Our review, which focuses on quantitative studies, corroborates earlier qualitative analyses^54^: according to the original authors’ encounter-level analyses, groups that are numerically larger than their opponents typically win BGCs within a given system, a pattern also observed in economic models.^92^^,^^93^ Our contribution here is to show that such “larger group advantage” conclusions are common across species (Figure 1), even though we could compare studies only on mean *absolute* group size and a binary indicator of whether relative size mattered. This indicates that groups are unlikely to face NPD dynamics during BGCs, as such situations would undermine cooperation unless additional mechanisms like reputation come into play.^18^^,^^94^ Instead, BGCs more closely align with biological and economic models that assume individual differences and asymmetries in benefit sharing, which promote within-group cooperation to the extent that differences in group size become a decisive factor in determining conflict outcomes.^52^^,^^95^

Monitoring individual contributions becomes increasingly challenging as overall group size increases, unless simple cues—such as presence or absence during a conflict—are used. Punishing non-contributors may be possible but requires detecting free riding, which becomes more difficult as group sizes and spatial spread increase. Two simpler mechanisms may therefore link individual contributions to eventual gains. One possibility is that contributions are state dependent, such that hunger increases both the motivation to participate in BGCs and the incentive to compete for the public good; supporting this, vervet monkeys prioritize high-reward food sites when contesting with group members,^96^ and human studies report hunger-induced shifts in cooperation in repeated public goods games.^97^ Another possibility is that access to the public good is regulated by a few high-ranking individuals who are strongly motivated to participate and grant low-ranking members tolerance in return for their contributions. This scenario aligns with empirical evidence across primate species showing that dominant individuals exchange tolerance or support for services such as grooming.^98^^,^^99^

### Potential explanations for why there is no consistent pattern in empirical data

Groups that are larger than their opponents benefit from numerical superiority, especially if dominant individuals gain disproportionately from access to the contested resource, as this promotes their participation. Vervet monkeys exhibit clear hierarchies that regulate priority of access to food,^85^ and dominant individuals generally participate more in BGCs. Adult females are considered co-dominant with males,^85^ while males may gain reputational benefits that translate into increased mating opportunities.^100^ Theoretical work shows that seemingly altruistic contributions to BGCs, including parochial altruism in humans, can be self-serving if contributors later benefit from elevated social status.^51^^,^^101^ However, our admittedly limited dataset on vervet monkeys indicates that larger groups (absolute size) are also generally more spatially spread (see larger groups have a larger group spatial spread section). A large group spread may offset the numerical superiority, as tightly clustered groups (lower group spatial spread) showed higher odds of winning BGCs. In contrast, more dispersed groups fared worse (see groups that are less spread are more likely to win section) (Figure S2). Although not statistically significant, NDVI at the encounter location—used as a proxy for food abundance^102^^,^^103^—suggests that incentives to compete are higher when potential rewards are higher (Figure S3). This implies that joint action may be more effective when more individuals have a stake in contested resources. Together, these findings indicate that both absolute group size and size asymmetries between opponents, as well as group spatial spread and seasonal ecology, jointly shape between-group contest dynamics, highlighting the need for further comparative studies across species beyond the semi-savanna, the highly omnivorous habitat of vervets.^104^^,^^105^

Building on the importance of coordination and group spatial spread, variability in empirical outcomes may arise from limits on patch sizes. When all group members cannot feed simultaneously in the same patch, as in a fruit tree, larger groups tend to split during foraging. Consequently, coming together for a contested resource becomes costlier in larger groups (absolute size): individuals must travel longer distances to join a BGC and forgo alternative activities such as foraging, resting, or socializing. In our model, we capture these effects in a single “coordination cost” term, which should be interpreted as the average per-capita cost of assembling a joint response, rather than as an identical cost experienced by all individuals. Some group members who are already near the contested patch will incur lower costs, whereas more distant individuals will incur higher costs; the model collapses this spatial heterogeneity into an expected mean cost as a tractable approximation. These opportunity costs reduce participation and, in turn, the odds of larger groups winning, a pattern consistent with our model predictions for vervet monkeys. Future studies should examine whether individuals initially distant from a BGC are less likely to join fights that ultimately succeed. If these individuals fail to access the won resource, they are better described as “loners” rather than cheaters/free riders.^106^ More generally, quantifying the total value of contested resources (e.g., patch size) may help explain variation in individual contributions, as optimality models predict higher participation when expected net payoffs are greater.

Evidence from other species shows that different ecological and social contexts can alter patterns of group success. In black-and-white colobus (guerezas; *Colobus guereza*), groups are smaller (4–11 individuals) than those of vervets (21–50 in our study, see Table S3), and BGC participation is male dominated, unlike female-dominated vervet groups.^83^^,^^84^^,^^85^ Here, outcomes depend on the dominant male’s body size, and additional males may be a liability if subordinates prioritize mating over fighting.^75^^,^^107^ Similarly, Verreaux’s sifakas (*Propithecus verreauxi*) show an intermediate pattern, where medium-sized groups win more often, but smaller and larger groups lose more frequently.^77^ Even within vervet monkeys, contests occurring further from core areas or in low-resource zones see reduced participation and winning outcomes,^83^ highlighting how local conditions influence individual decisions to engage.^108^ Our vervet-inspired model shows that limited food patch size (that may vary across seasons and according to local resource availability) predicts how widely spatially spread groups are, thus affecting who turns up to fight. Indeed, larger groups may fail to capitalize on their numerical advantage when the costs of leaving profitable patches and assembling are high, and mean payoffs of winning the BGC itself are diluted if acquired patches do not feed all group members. As a consequence, relatively larger groups may assemble smaller actual fighting groups at the contested resource and therefore become likely to lose because of lower fighting power and because assembled individuals avoid the risk of being injured. By contrast, as long as coherent group movements are not constrained by small food patches, larger groups appear to win consistently without obvious declines in participation.^109^^,^^110^ In these cases, the benefits of numerical advantage may outweigh the costs of within-group competition, illustrating that the success of large groups depends on both ecology and the way social and physiological systems distribute the costs of feeding and coordination.

### Insights from the mathematical model

Our model assumed that all individuals within a group have equal access to the resource gained after a successful BGC. This assumption gives the smaller group (relative terms) in a contest an advantage because the mean gains will be higher. Accordingly, the model reveals a parameter space—especially when patch capacity is small relative to groups’ absolute (census) sizes—in which the (relative) smaller group is more likely to win contests against the larger group (Figure 4F, right side of the gray vertical line), while for other parameter combinations the usual larger group advantage is recovered. This result is consistent with previous theoretical models showing that, within a contest, equal sharing of resources among group members gives the group with fewer individuals than its opponent a competitive advantage over the case in which only individuals who contributed to between-group competitions can access the resources.^92^^,^^95^ In the latter case, public goods essentially become private goods, making larger groups less susceptible to free riding and more likely to win BGCs. A natural extension would be to combine coordination costs with private goods. However, such a modeling approach would greatly benefit from empirical data that take into account, for example, how stronger individuals are spread over patches and how that in turn affects the probability that those who could monopolize the gained resource afterward actually join the BGC.

### Limitations of the study

Our study cannot determine whether resources alone fully explain how ecological conditions shape BGCs. Future work should incorporate finer scale measures—such as resource quality, spatial distribution, and seasonal variation—to better capture their influence on individual participation and contest outcomes. Coordination costs due to food patches being too small to accommodate entire groups may limit larger groups’ ability to act collectively, and tracking peripheral individuals could clarify the benefits of non-participation. Given the small vervet dataset and potential differences of the smallest group (AK), interpretations should be cautious. Here, the data primarily serve to motivate our modeling framework, emphasizing how coordination costs—especially those imposed by patch size—affect contest outcomes. Our cross-species literature review is therefore not a direct quantitative test of the model, because we lack contest-level data on group sizes and patch sizes for most systems. Instead, the model is intended as a mechanistic illustration of how, within a given species or population, the interplay between absolute group size, patch capacity, and coordination costs can sometimes favor smaller groups, as in our vervet case study. Additionally, although all data were collected from the same population, different questions drew on observations from slightly distinct time periods (see Table S3).

### Conclusions

By combining a systematic review, empirical analyses, and a simple mathematical model, we show how smaller groups (relative terms) can sometimes prevail in BGCs despite the general advantage of larger groups. The model provides a mechanistic explanation: coordination costs arising from group spatial spread can give smaller groups a relative advantage. This framework highlights measurable ecological variables—such as patch size and group dispersion—that future studies should quantify to test the model’s assumptions. By linking resource-driven group structure to participation decisions, our approach underscores the importance of incorporating ecological realism into theoretical models of cooperation and offers a foundation for broader analyses of N-player cooperation in both non-human and human systems.

## Resource availability

### Lead contact

Any information or requests involving data resources and methods can be addressed to the lead contact, Miguel Gareta Garcia (miguelgaretagarcia@gmail.com).

### Materials availability

This study did not generate any physical materials.

### Data and code availability

• All the data reported in this paper are provided in the supplemental information file.
• All the original code has been deposited at the Open Science Framework (OSF) and is publicly available. The DOI is listed in the key resources table.
• Any information concerning data, analysis of data, or modeling part that is included in this paper is available from the lead contact upon request.

## Acknowledgments

X.-Y.L.R. is supported by the 10.13039/501100001711Swiss National Science Foundation (SNF) Starting Grants (211549). The study was in part financed by 10.13039/501100001711SNF grants 310030B_173334/1 and CRS133_133040 to R.B. The field site for the case study was funded by 10.13039/501100001711SNF grants P300P3_151187, 31003A_159587, and PP00P_3170624, along with a Branco Weiss Fellowship–Society in Science grant to E.v.d.W. M.G.G. was supported during data analyses by 10.13039/501100001711SNF grant PP00P3_198913 to E.v.d.W. At the time of writing, E.v.d.W. was supported by the 10.13039/501100000781European Research Council under the 10.13039/501100007601European Union’s Horizon 2020 research and innovation program for the 10.13039/100017325ERC “KNOWLEDGE MOVES” starting grant (grant agreement no. 949379), and the Mind Brain Behavior fellowship and Human Evolutionary Biology Department at Harvard University supported M.G.G. We thank the indispensable contributions of many researchers who participated in the habituation and data collection at the IVP study site from 2011 to 2019, especially Dr. Jean Arseneau-Robar, who designed the data collection protocols for BGCs. We are also grateful to the managers at the study site, Albert Driescher and Arend van Blerk, as well as the van der Walt family, for allowing our research activities on their land. Additionally, we appreciate the support of Ezemvelo KZN Wildlife for approving the conduct of our activities in South Africa. Further, we appreciate the correspondence with Prof. Bonaventura Majol, Prof. Christina Riehl, Prof. Michelle Brown, Prof. Meg Crofoot, Prof. Julie Teichroeb, and Dr. Claudia Fichtel, who kindly shared valuable information on group sizes during our literature review. Finally, we are grateful to Dr. Radu Slobodeaunu from the University of Neuchâtel for his insights on statistical analysis.

## Author contributions

M.G.G., R.B., and X.-Y.L.R. conceived the study, X.-Y.L.R. and R.B. conceived the modeling part, and X.-Y.L.R. developed it. M.G.G. analyzed the data, and E.v.d.W. provided the data for the vervet monkey case study that were collected by the IVP team of researchers, including post-docs, PhDs, MSc students, and research assistants. M.G.G. and R.B. wrote the manuscript, and X.-Y.L.R., R.B., E.v.d.W., and M.G.G. worked together on the different edits of the manuscript.

## Declaration of interests

The authors declare no competing interests.

## STAR★Methods

### Key resources table

**Table:** 

REAGENT or RESOURCE	SOURCE	IDENTIFIER
**Deposited data**		

Data	Open Science Foundation (OSF)	https://osf.io/g6789/
Code	Open Science Foundation (OSF)	https://osf.io/g6789/

**Experimental models: Organisms/strains**		

Vervet monkeys *(Chlorocebus pygerythrus)*	INKAWU Vervet Project (IVP), Mawana Game Reserve, Kwa-Zulu Natal, South Africa	N/A

**Software and algorithms**		

R	N/A	https://cran.r-project.org/(v4.0.3)
Mathematica	Wolfram Research Inc.	https://www.wolfram.com/mathematica/v.14.2

### Method details

#### Literature review on animal between-group conflicts (BGCs)

Following the PRISMA flow diagram guidelines for systematic reviews,^111^ we first identified the literature using the Google Scholar search engine. We searched for articles containing the key words: (i) “inter-group”, “between-group”, “conflict”, or “encounters”, combined with (ii) “animal” and “wild”, and (iii) “win”, “lose”, or “outcome”, and (iv) “group size”. All steps of the literature review are shown in Figure S4, with a summary in Table S1. In the first screening step, this search yielded *N* = 395 records. In the second screening step, after reviewing the abstracts of all 395 papers, we identified 282 publications that met the basic inclusion criteria. In the third step of the eligibility process, we reviewed the methods and results sections of these publications and identified 32 studies reporting outcomes from wild-animal between-group encounters. In a fourth eligibility step, we added 7 additional references from the bibliographies of these 32 articles that also met our criteria.

For each of these 39 studies, we then extracted two study-level variables: (a) the reported mean total group size for the focal population, and (b) the authors’ quantitative conclusion on whether contest outcome depended on relative group size (i.e., whether, across the encounters they analyzed, larger groups tended to win more often or not). Finally, after excluding studies that did not provide all these elements, we retained a final sample of *N* = 33 studies, each of which reported (a) the average total group size, (b) a clear conclusion regarding whether larger groups won more frequently, and (c) a quantitative statistical analysis explicitly relating winning outcomes to relative group size (i.e., whether the numerically larger or smaller group in a contest tended to win), even though detailed, contest-by-contest group sizes were rarely provided. Because such detailed group-size data were usually unavailable, our review necessarily treats each study as a single binary datapoint (‘larger group wins more often’ versus ‘not’), providing only a coarse summary of the literature.

#### Ethic statement

The study of vervet monkeys across years complied with the Association for the Study of Animal Behavior Guidelines for the Use of Animals in Research. Data collection consisted solely of observations of individuals habituated to human presence and did not involve handling or experimental manipulation. Vervet monkeys are not a protected species in South Africa, and observational research was conducted at a private reserve owned by the van der Walt family, which granted permission for our work. Further, the study was approved by the relevant local authority, Ezemvelo KZN Wildlife.

#### Study of BGCs in vervet monkeys

Despite not being territorial, vervet monkeys provide an excellent study system because BGCs are frequent and non-lethal.^78^^,^^112^^,^^113^ Across more than a decade of observation at this site, and in previous detailed studies of this population, severe injuries and fatalities during BGCs have not been reported; instead, the prevailing costs appear to be energetic, opportunity, and occasional non-lethal injuries (personal observations by E.v.d.W., R.B., and M.G.G.). We used quantitative data from three habituated wild groups at the INKAWU Vervet Project (IVP) in Mawana Game Reserve, KwaZulu-Natal, South Africa. These datasets were then used to examine the associations described below. Different IVP researchers collected all datasets after receiving training from senior researchers and passing individual-identity and data-collection tests, with an inter-observer agreement of 80% or higher. In all empirical analyses reported here, group-level predictors (group size, group spatial spread) are treated as absolute values for the focal group in each contest, not measured relative to the opponent. While the response variable (winning) is inherently a contest outcome, our main statistical models do not include the opponent’s group size or group spatial spread as predictors (i.e., we do not use size differences or size ratios).

##### Association between total group size and group spatial spread

Group spatial spread is the spatial extent of the group at a given moment, measured as the maximum distance between any two peripheral individuals. For each observation, we recorded the estimated locations of all group members (all age classes). This approach quantifies the group’s spatial distribution at a given time, ensuring that the resulting value reflects the group’s spread at that moment. This measure is based on individual focal sampling, in which a specific individual is followed for 20 min while tailored behavioral information is recorded on handheld devices. The behaviors collected during focal animal sampling included information about the focal individual’s activity (foraging, moving, resting, social), GPS location, position in the group (center, periphery, head, back), and group spatial spread. Our focal data recorded the spatial spread of the group at the beginning and end of each focal, which could fall into the following categories: (i) 0–20 m, (ii) 20–50 m, (iii) 50–100 m, (iv) > 100 m, (v) across the river, and (vi) unknown whenever the data collector was not certain (e.g., after river crossings or rapid activity changes due to human disturbance). Of these six possible categories, we excluded “across the river” because of the difficulty in accurately assessing the presence of all individuals. Accordingly, we retained four distance-based group spatial spread categories and assigned them numerical labels as follows: 0–20 m = 1, 20–50 m = 2, 50–100 m = 3, and >100 m = 4 (Figure S5). Following Canteloup et al. (2019), we included season as a factor in our analyses to account for the pronounced seasonality in the study area. We defined “winter” as the dry, colder period with lower food availability, from 1 May to 31 October, and “summer” as the remainder of the year.^114^ Seasonal changes are likely to influence patterns of group spatial spread, as individuals may need to disperse more widely when food is scarce during the dry season, which, in turn, could affect BGC outcomes.

##### Association between group spatial spread and winning odds

To evaluate the effect of group spatial spread on contest outcomes, we analyzed BGCs for which we had information on (i) both the focal and rival groups’ spatial spread and (ii) the encounter outcome, which was either won or not won by the focal group. Because these data were collected by a researcher following one of the two groups involved in the BGC, the focal group is the group being followed, and the other is the rival group. Encounters in which both groups had identical group spatial spread were excluded from analysis. The season was determined by the date of the encounter, following previous studies on the same population: winter (drier, colder conditions from 1 May to 31 October) and summer (warmer conditions with seasonally higher but variable rainfall during the remainder of the year).^114^^,^^115^ A group was classified as the winner if it actively displaced its rival from the contest location, consistent with criteria used in recent studies.^78^ As a proxy for ecological value and food abundance, we included NDVI (Normalised Difference Vegetation Index)^102^^,^^103^ values from our previous publication, calculated as the NDVI of the quadrat where the BGC occurred relative to the mean annual NDVI of the focal group’s home range.^83^ Accordingly, each BGC had an NDVI value obtained from satellite imagery, with an average lag of 7.54 ± 8.66 days between image acquisition and the BGC. Our dataset included a total of 71 BGCs across different group-dyad combinations: 34 for AK–BD, 16 for AK–NH, 10 for BD–CR, and 11 for BD–NH (order not descriptive of which was the focal group).

### Quantification and statistical analysis

#### Description of literature review on animal BGCs

In this section, we provide descriptive statistics and graphical representations illustrating the frequency of winning in relation to whether groups were larger than their opponents. Data extracted from 33 published studies enabled us to summarise overall patterns of group-level outcomes, showing how often the numerically larger group in a contest won over the smaller one. However, due to limited detail in the literature—particularly the lack of information on the absolute sizes of each group in specific dyadic encounters—we were unable to perform more robust statistical analyses. We therefore focused on descriptive summaries to highlight general trends.

#### Study of BGCs in vervet monkeys

##### Model I. Association between total group size and group spatial spread

To reduce autocorrelation in the spatial data,^116^^,^^117^^,^^118^^,^^119^ we included only one measurement taken at the beginning of each individual focal follow. The final dataset comprised 5,303 data points from the four group spatial spread categories (0–20 m, 20–50 m, 50–100 m, and >100 m) across three vervet monkey groups (AK: 792 data points, 15.17%; NH: 2,003, 38.36%; BD: 2,427, 46.47%), with average group sizes of AK = 21.32 ± 1.5, NH = 34.04 ± 4.88, and BD = 50.58 ± 2.64 individuals. To analyze the ordinal response variable of group spatial spread, we used a cumulative link mixed model (CLMM) implemented with the clmm function in the ordinal package^120^ in R.^121^ The model assumes an ordinal error distribution with a logit link function and equidistant thresholds between categories. Total group size, season (summer/winter), and their interaction were included as fixed effects, and focal individual and date were included as random effects to account for repeated measures and temporal structure. We compared an additive model with main effects of total group size and season to an interaction model using AIC and a likelihood ratio test because the interaction model fit substantially better (ΔAIC = 31.8; χ^2^_1_ = 33.8, *p* < 0.001).

##### Model II. Association between group spatial spread and winning odds

The total group sizes for the four different groups included in this analysis were as follows: AK = 22.14 ± 3.72, NH = 36.08 ± 8.37, BD = 50.91 ± 3.34, and CR = 40.60 ± 5.87 individuals. To evaluate the effects of group spatial spread, season, and local resource abundance (NDVI) on the likelihood of winning between-group contests, we employed a Bayesian generalized linear mixed modeling approach using the brms package^122^ in R.^121^ We modeled the response variable of binary contest outcome (FocalWins: win/lose) as a function of group spatial spread difference (calculated as the focal group’s spread mean at the encounter time minus that of its rival’s), season (summer/winter), and the NDVI value at the BGC location relative to the mean annual NDVI of the focal groups’ home range as predictors. To account for repeated measures and group-level dependency, we included a random intercept for the four possible group dyads. We compared an additive model (including only main effects) to a model including all two-way interactions among predictors. Both models were fitted with Bernoulli likelihoods and logit link functions, using four Markov Chain Monte Carlo (MCMC) chains of 2,000 iterations each (1,000 for warm-up). Model selection was based on leave-one-out cross-validation information criterion (LOOIC), with the model exhibiting the lower LOOIC deemed to have superior predictive performance. The additive model (without interactions) was preferred, as the interaction terms did not improve predictive fit (ΔELPD = 6.9, SE = 7.5). For this final model, we summarised fixed effects by their posterior means and 95% credible intervals, obtained from the marginal posteriors (Table S4).

#### Mathematical model

##### Mathematical model exploring group size-dependent coordination cost on the outcome of BGCs

In this model, we consider the fact that group-living animals need to coordinate to organise around a BGC. The cost of coordination increases with group size and group spatial spread. We model this using a multi-phase sigmoid function

(Equation 1) $$c_{g}=\sum_{i=1}^{m}\frac{C_{i}}{1+e^{-k_{i}\left(n-n_{i}\right)}}\text{,}$$

where *n* represent the group size, *C*_*i*_ is the magnitude of the cost increase at transition *i* (e.g., in the vervets, the cost of recruiting group members from the tree *i*), *k*_*i*_ controls how steep the transition is (corresponding to how hard it is to recruit the first individual from the tree *i* compared to getting the rest of the individuals from the same tree to join), *n*_*i*_ is the center of transition *i* (the middle point of each S-shaped step increases), and *m* is the number of steps (e.g., the number of fruit trees where group member clusters are distributed). In Figure 4A, the parameter values were set to *m* = 4, and for all *i* = 1,2,3,4, *C*_*i*_ = 5,*k*_*i*_ = 1, *n*_*i*_ = 10*i*.

The expected coordination cost for an individual group member (Figure 4B) is then

(Equation 2) $$c_{a}=c_{g}/n\text{.}$$

Here, *c*_*g*_ should be interpreted as the *group-level* coordination cost and *c*_*a*_ as the *average per-capita* cost of mobilising the group for a BGC. In practice, individuals closer to the contested resource will incur lower costs than those further away, but we approximate this spatial variation by its mean across group members to keep the model analytically tractable. Coordination costs are expected to increase with group spatial spread, because individuals that are farther from the BGC location face higher energetic and injury risks if they join, can instead exploit alternative resources with reduced within-group competition, and may be less exposed to social incentives or sanctions (e.g., punishment by high-ranking group members) that promote participation.^112^

The expected gain from a BGC (e.g., the amount of food) in small groups can often be limited by the maximum consumption capacity of all group members to reach satiation. For example, a lion pride will usually be unable to consume an entire wildebeest. However, in large groups, the benefits of a BGC may not be sufficient for everyone, as seen in the vervet monkeys we study. Therefore, we model the expected group gain as a piecewise function that first increases with group size and then levels off.

(Equation 3) $$g_{g}=\mathrm{Min}\left(sn,v\right)\text{,}$$

where *s* represents the average consumption capacity of each group member, and *v* stands for the value of the contested resource.

#### The expected gross gain for an individual group member is

(Equation 4) $$g_{a}=g_{g}/n\text{.}$$

In Figure 4C, we set *s* to 1 and *v* to 10.

The expected net gain for an individual group member is calculated by subtracting the expected individual coordination cost from the expected gross gain from a successful BGC (Figure 4D),

(Equation 5) $$g=g_{a}-c_{a}\text{.}$$

We then model an individual’s probability of participating in a BGC to be proportional to the expected net gain, when the gain is positive (Figure 4E):

(Equation 6) p=Max(0,ag),

where *α*∈(0, 1) adjusts the general motivation to participate in a BGC.

#### The realised fighting group size is then

(Equation 7) S=np.

that is, the product of total group size and the probability that an individual participates (Figure 4F). Finally, we assume that contest success depends on this realised fighting group size rather than on census size per se, so that, all else equal, the group with the larger *S* is more likely to win a BGC.
