# Supplementary material for: Coordination costs may counteract the positive effects of a larger group size in determining the winner of between-group conflicts
Source: iScience. 2026 Mar 30;29(5):115496. doi: 10.1016/j.isci.2026.115496 (PMC13129389; doi:10.1016/j.isci.2026.115496)
Supplement: Document S1. Figures S1–S5 and Tables S1–S4 [file mmc1.pdf]

iScience, Volume *29*

Supplemental information

Coordination costs may counteract the positive effects of a larger group size in determining the winner of between-group conflicts

Miguel Gareta García, Erica van de Waal, Xiang-Yi Li Richter, and Redouan Bshary

Supplementary Information for: Coordination costs may counteract the positive effects of a larger group size in determining the winner of between-group conflicts

Miguel Gareta García, Erica van de Waal, Xiang-Yi, Li Richter, Redouan Bshary[2,3]

Number reference	Name species	Contest type	Average total group size	Larger group wins	Reference
1	Barnacle geese	resources	3.50	1	[1]
2	Grey wolf	territory	8.70	1	[2]
3	Free-ranging dogs	territory	7.00	1	[3]
4	Ethiopian wolf	territory	6.10	1	[4]
5	Capuchin monkeys	territory	18.75	1	[5]
6	White-faced capuchin monkeys	territory	24.50	1	[6]
7	Wedge-capped capuchin monkeys	resources	19.90	1	[7]
8	Blue monkeys	territory	29.45	1	[8]
9	Vervet monkey	resources	39.80	0	[9]
10	Guerezas	resources	29.80	0	[10]
11	Ursine colobus monkeys	resources	21.25	1	[11]
12	Spotted hyena	territory	47.00	1	(Hofer & East, 1993)
13	Grey-cheeked mangabeys	resources	8.50	1	[13]
14	Rhesus monkeys	resources	22.00	1	[14]
15	Lion	territory	17.00	1	[15]
16	Lion	territory	15.00	1	[16]
17	Yellow baboon	resources	29.60	1	[17]
18	Chacma baboons	resources	38.50	1	[18]
19	Verreaux's Sifaka	territory	6.30	0	[19]

Number reference	Name species	Contest type	Average total group size	Larger group wins	Reference
20	Verreaux's Sifaka	territory	6.50	1	[20]
21	Verreaux's Sifaka	territory	4.62	0	[21]
22	Tufted capuchin monkeys	territory	17.37	1	[22]
23	Tufted capuchin monkeys	resources	24.50	0	[23]
24	Meerkats	territory	20.00	1	[24]
25	Ring-tailed lemur	territory	15.5	0	[25]
26	Banded mongoose	territory	11	1	[26]
27	Banded mongoose	territory	18	1	[27]
28	Japanese macaques	resources	48.7	1	[28]
29	Green woodhoopoe	territory	5.6	1	[29]
30	Vervet monkeys	resources	43	1	[30]
31	African wild dogs	territory	7	1	[31]
32	Tasmanian hen	territory	5.5	0	[32]
33	Mountain gorillas	resources	9.5	0	[33]

Table S1. Literature review with information about species in common name, contest type, average group size, larger group winning or not (1 equal to yes, 0 equal to no), and publication reference (Related to Figure 1).

Data used	Factors	Hypotheses	Predictions
Vervet monkey empirical data	Group spatial spread and relative group size	Group size affects how individuals are spatially distributed	Larger groups are more spatially spread than smaller groups
	Group spatial spread, season, and NDVI	Group spatial distribution mediates coordination and affects winning odds	Groups that are more spatially spread are less likely to win between-group conflicts (BGCs)

Table S2. Overview of empirical analyses conducted on vervet monkey data, the factors motivating each analysis, and the associated hypotheses and predictions.

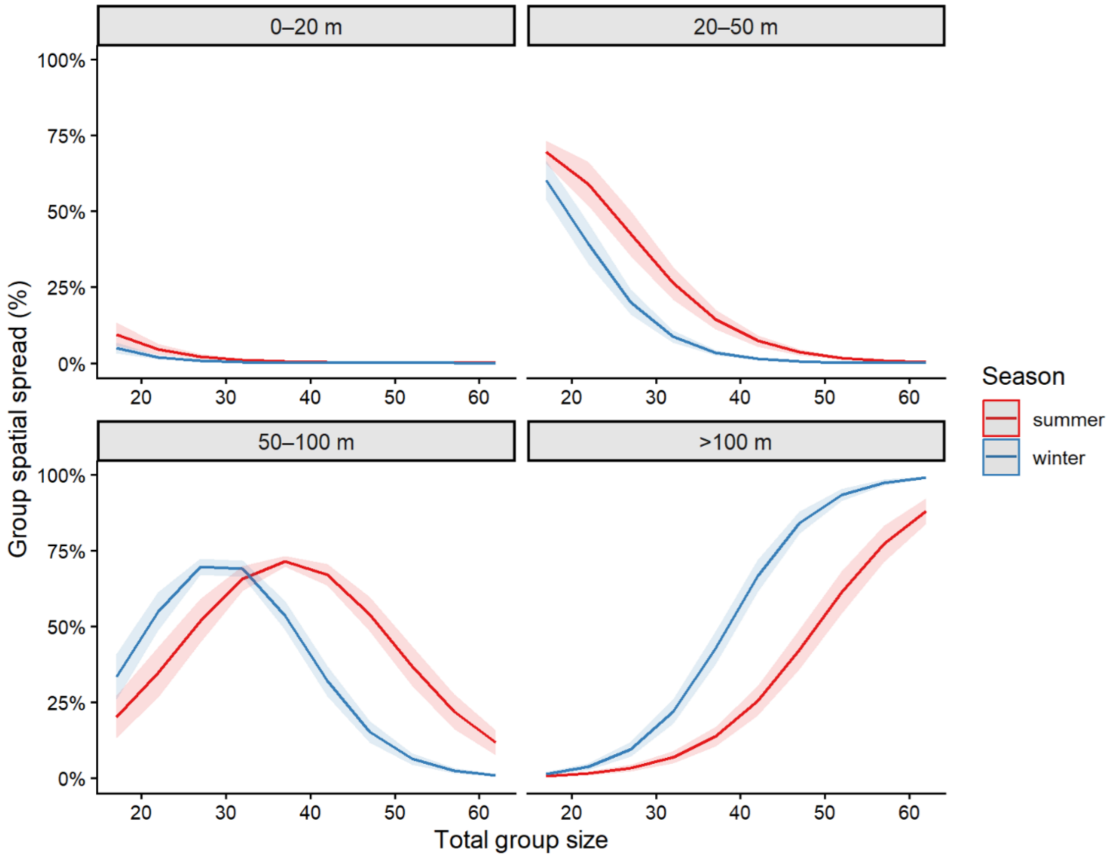

Figure S1. Model-predicted probabilities of group spatial spread categories as a function of total group size and season for three vervet groups (AK, NH, BD). Panels show the predicted probability that a vervet group falls into each spatial spread category (0–20 m, 20–50 m, 50–100 m, >100 m) across the observed range of total group sizes, based on the cumulative link mixed model (Model I) fitted to data from groups AK, NH, and BD. Red lines and bands indicate summer, blue lines and bands indicate winter; shaded areas show 95% confidence intervals. Small spatial spreads (0–50 m), indicating greater spatial cohesion, become less likely as group size increases, whereas very large spatial spreads (>100 m) become more likely, particularly in winter.

		Total group size		N adult females		N adult males	
**(A) Group Size and Group Spatial Spread**	**Group**	**Mean**	**SD**	**Mean**	**SD**	**Mean**	**SD**
Period study: Mar2016-Jan2019	AK	21.31	1.58	4.13	0.38	2.44	0.89
	NH	34.03	4.88	7.75	1.57	2.72	1.37
	BD	50.58	2.63	14.6	1.48	10.24	3.81
		**Total group size**		**N adult females**		**N adult males**	
**(B) Group Spatial Spread and Winning BGCs**	**Group**	**Mean**	**SD**	**Mean**	**SD**	**Mean**	**SD**
Period study: Jan2016-September 2019	AK	22.14	3.72	6.81	1.20	2.47	0.75
	NH	36.08	8.37	11.16	3.62	2.6	1.25
	BD	50.91	3.34	20.95	4.69	6.79	3.45
	CR	40.60	5.87	12.40	2.27	7.90	1.28

Table S3. Group sizes and study periods for the three empirical analyses. For each block—(A) group size and group spatial spread, and (B) group spatial spread and winning BGCs—we report the mean (± s.d.) total group size and the numbers of adult females and adult males for each group. Analyses (A) includes three vervet groups (AK, NH, BD), whereas analysis (B) includes a fourth group (CR) because this group contributed BGCs with spatial-spread and outcome data during 2016–2019.

**MODEL GROUP SPATIAL SPREAD, SEASON, NDVI AND WINNING ODDS**	**Posterior Mean**	**95% CI**
brm(FocalWins ~ Group spatial spread difference + Season + relative_ndvi_to_HR_during_IGE + (1 | DyadGroup2), family = bernoulli(link = "logit"), data = data_glmm_ndvi)		
Group spatial spread difference	-0.52	-0.94 to -0.12
Season (Winter vs. Summer)	-0.51	-1.78 to 0.67
Relative NDVI at encounter location	0.00	-0.02 to 0.00

Table S4. Posterior mean estimates and 95% credible intervals from the Bayesian generalised linear mixed model (additive structure, no interactions) predicting the probability of the focal group winning BGCs. Negative values of group spatial spread difference (from the perspective of the focal) indicate greater focal group spatial cohesion – or less spatial spread – relative to the rival. Only spatial spread difference had a credible effect on win probability; the effects of season and relative NDVI were not statistically significant, although both showed a tendency toward lower win probability in winter and at lower NDVI. This model was fitted to contests among four vervet groups (AK, NH, BD, CR) (Related to Figure S2 and S3).

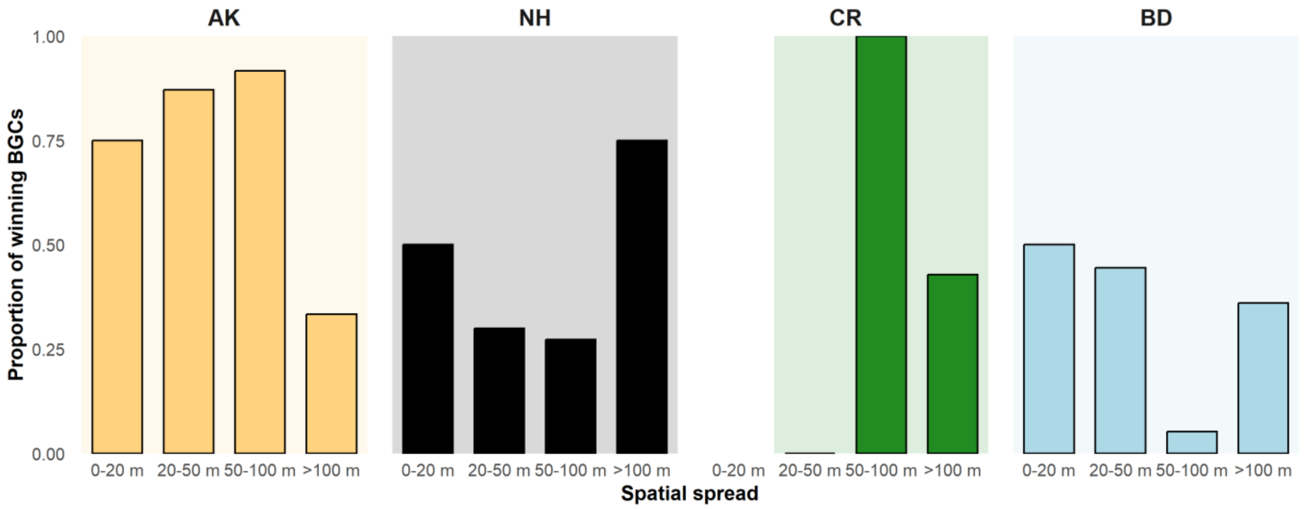

Figure S2. Proportion of winning and differential group spatial spread for the four vervet groups included in the winning-odds analysis (B): AK (light orange), NH (grey), CR (green), and BD (yellow). Along the y-axis is the proportion of BGCs the group won against other possible groups, expressed as a percentage (1 = 100%). Along the x-axis is the differential spatial group spread from the focal group's perspective relative to its rival, categorised as less spatial spread (< Spread), same spatial spread (Same), or more spatial spread (> Spread). The group CR has a smaller sample size due to an interruption in the monitoring (Related to Table S4 and Figure S3).

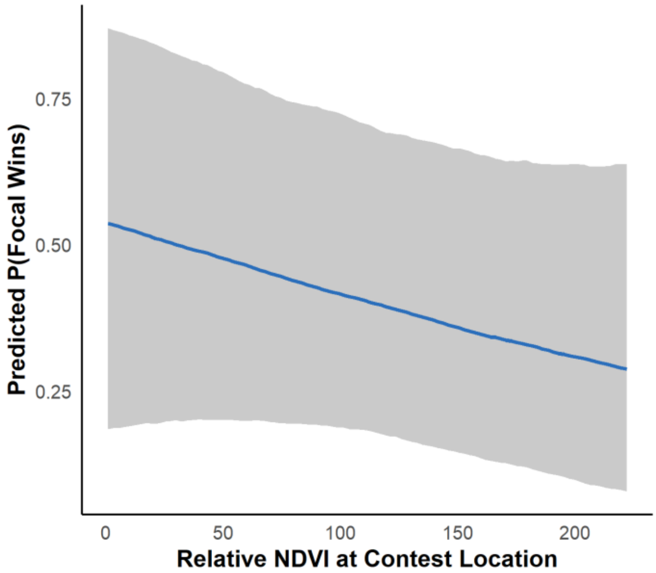

Figure S3. Posterior predictions from the Bayesian model for the effect of relative NDVI at the contest location on the probability that the focal group wins, based on contests among four vervet groups (AK, NH, BD, CR). The line represents the posterior mean and the shaded ribbon the 95% credible interval (Related to Table S4 and Figure S2).

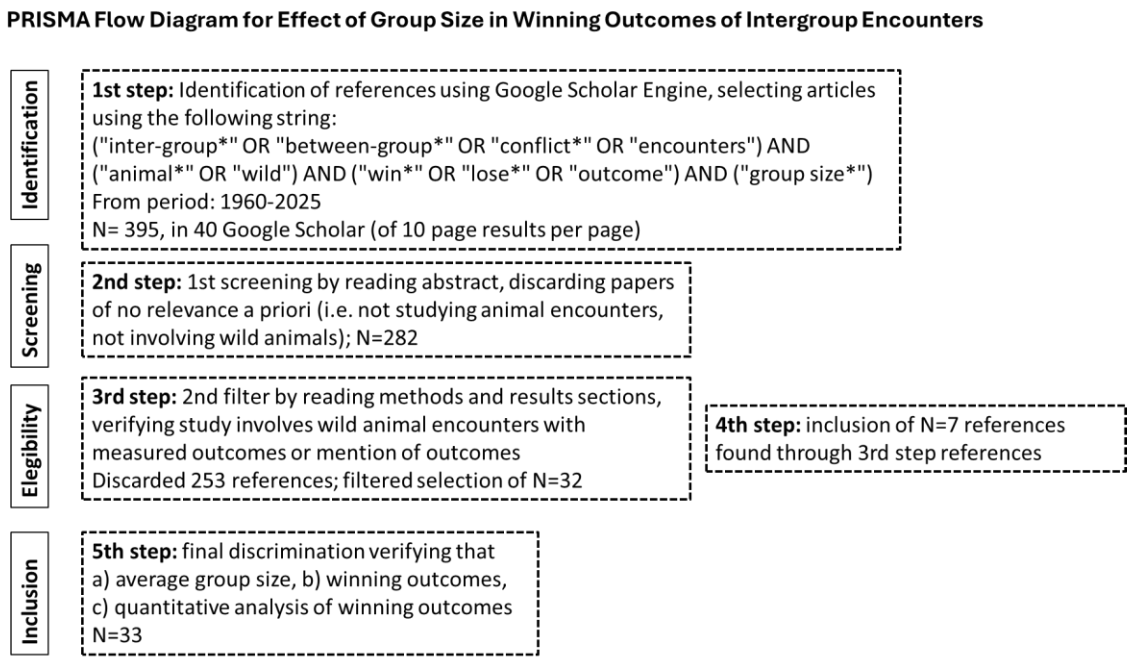

Figure S4. Flow diagram following the meta-analysis approach of Majolo et al. 2020 [34] and the PRISMA guidelines [35]. From top to bottom, the steps describe how to identify, screen, select, and include the various references. More details on the criteria used for the targeted literature review papers are provided in the Methods section (Related to Figure 1).

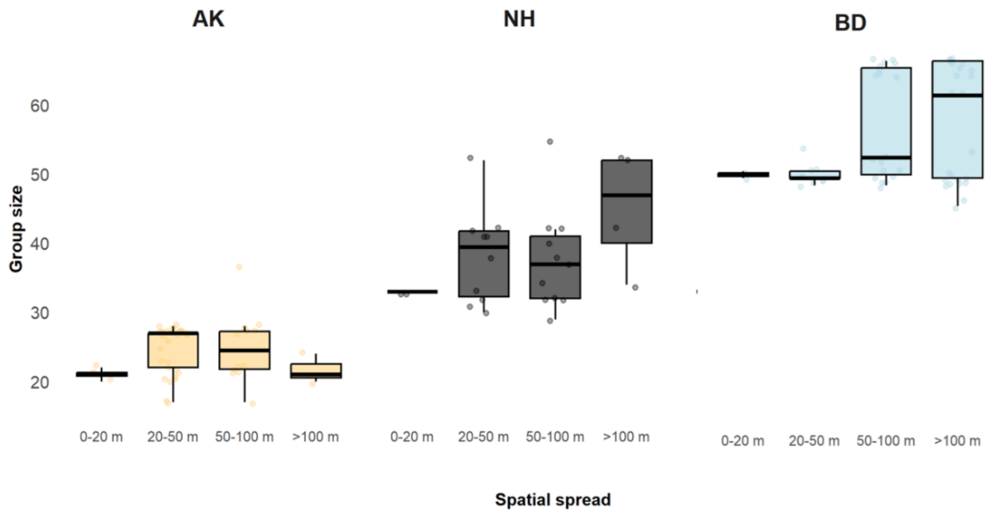

Figure S5. Graph representing the group spatial spread distribution for the three vervet groups used in the spread analyses (from smallest to largest): AK in light orange, NH in grey, and BD in blue. Shown along the x-axis are the different group spatial spread categories: 0–20 m, 20–50 m, 50–100 m, and >100 m. The boxplots represent the median and interquartile range of group size for each group within each group spread category. This dataset covers the years 2016–2019 (related to Figure 2).

Supplementary references

Arseneau-Robar, T. J. M., Taucher, A. L., Müller, E., van Schaik, C., Bshary, R., & Willems, E. P. (2016). Female monkeys use both the carrot and the stick to promote male participation in intergroup fights. *Proceedings of the Royal Society B: Biol. Sci. 283*(1843). https://doi.org/10.1098/rspb.2016.1817

van Belle, S., & Scarry, C. J. (2015). Individual participation in intergroup contests is mediated by numerical assessment strategies in black howler and tufted capuchin monkeys. *Philosophical Transactions of the Royal Society B: Biol. Sci. 370*(1683). https://doi.org/10.1098/rstb.2015.0007

Benadi, G., Fichtel, C., & Rappeler, P. (2008). Intergroup relations and home range use in Verreaux's sifaka (Propithecus verreauxi). *Am. J. Primatol.*, *70*(10), 956–965. https://doi.org/10.1002/ajp.20588

Black, J. M., & Owen, M. (1989). *Agonistic behaviour in barnacle goose flocks: assessment, investment and reproductive success. Anim. Behav. 37, 199-209*.

Bonanni, R., Natoli, E., Cafazzo, S., & Valsecchi, P. (2011). Free-ranging dogs assess the quantity of opponents in intergroup conflicts. *Anim. Cognition*, *14*(1), 103–115. https://doi.org/10.1007/s10071-010-0348-3

Brown, M. (2020). Detecting an Effect of Group Size on Individual Responses to Neighboring Groups in Gray-Cheeked Mangabeys (Lophocebus albigena). *Int. J. Primatol. 41*(2), 287–304. https://doi.org/10.1007/s10764-020-00144-9

Cant, M. A., Otali, E., & Mwanguhya, F. (2002). Fighting and mating between groups in a cooperatively breeding mammal, the banded mongoose. *Ethology*, *108*(6), 541–555. https://doi.org/10.1046/j.1439-0310.2002.00795.x

Cassidy, K. A., MacNulty, D. R., Stahler, D. R., Smith, D. W., & Mech, L. D. (2015). Group composition effects on aggressive interpack interactions of gray wolves in Yellowstone National Park. *Behav. Ecol. 26*(5), 1352–1360. https://doi.org/10.1093/beheco/arv081

Cowlishaw, G. (1995). Behavioural Patterns in Baboon Group Encounters: The Role of Resource Competition and Male Reproductive Strategies. *Behaviour*, *132*(1), 75–86.

Crofoot, M. C., Gilby, I. C., Wikelski, M. C., & Kays, R. W. (2008). Interaction location outweighs the competitive advantage of numerical superiority in Cebus capucinus intergroup contests. *Proc. Natl. Acad. Sci. USA 105*(2), 577–581. https://doi.org/10.1073/pnas.0707749105

Dyble, M., Houslay, T. M., Manser, M. B., & Clutton-Brock, T. (2019). Intergroup aggression in meerkats. *Proceedings of the Royal Society B: Biol. Sci. 286*(1917). https://doi.org/10.1098/rspb.2019.1993

Pride, R.E., Felantsoa, D., Randriamboavonjy, T., & Randriambelona, R. (2006). Resource Defense in Lemur catta: The Importance of Group Size. In A. Jolly, Sussman R. W., Koyama N., & Rasamimanana H. (Eds.), *Ringtailed lemur biology: Lemur Catta in Madagascar* (pp. 208–232). Springer, New York.

García, M. G., De Guinea, M., Bshary, R., & van De Waal, E. (2022). Drivers and outcomes of between-group conflict in vervet monkeys. *Philosophical Transactions of the Royal Society B: Biol. Sci. 377*(1851). https://doi.org/10.1098/rstb.2021.0145

Harris, T. R. (2010). Multiple resource values and fighting ability measures influence intergroup conflict in guerezas (Colobus guereza). *Anim. Behav. 79*(1), 89–98. https://doi.org/10.1016/j.anbehav.2009.10.007

Hofer, H., & East, M. L. (1993). *The commuting system of Serengeti spotted hyaenas, how a predator copes with migratory prey. II. Intrusion pressure and commuters' space use*. *Oecologia 3*(46), 547–557.

Johnstone, R. A., Cant, M. A., Cram, D., & Thompson, F. J. (2020). Exploitative leaders incite intergroup warfare in a social mammal. *Proc. Natl. Acad. Sci. USA*, *117 (47)*, 202003745. https://doi.org/10.1073/pnas.2003745117

Jordan, N. R., Buse, C., Wilson, A. M., Golabek, K. A., Apps, P. J., Lowe, J. C., van der Weyde, L. K., & Weldon McNutt, J. (2017). Dynamics of direct inter-pack encounters in endangered African wild dogs. *Behav. Ecol. Sociobiol. 71*(8). https://doi.org/10.1007/s00265-017-2338-9

Koch, F., Signer, J., Kappeler, P. M., & Fichtel, C. (2016). The role of the residence-effect on the outcome of intergroup encounters in Verreaux's sifakas. *Sci. Rep.*, *6*(June), 1–7. https://doi.org/10.1038/srep28457

Lewis, R. J., Sandel, A. A., Hilty, S., & Barnett, S. E. (2020). The Collective Action Problem but Not Numerical Superiority Explains Success in Intergroup Encounters in Verreaux's Sifaka (Propithecus verreauxi): Implications for Individual Participation and Free-Riding. *Int. J.Primatol. 41*(2), 305–324. https://doi.org/10.1007/s10764-020-00155-6

Majolo, B., deBortoli Vizioli, A., Martinez-Iñigo, L., & Lehmann, J. (2020). The effect of group size and individual characteristics on between-group encounters in primates. *Int. J.Primatol. 41*(2), 325–341.

Markham, A. C., Alberts, S. C., & Altmann, J. (2012). Intergroup conflict: Ecological predictors of winning and consequences of defeat in a wild primate population. *Anim. Behav. 84*(2), 399–403. https://doi.org/10.1016/j.anbehav.2012.05.009

Meunier, H., Molina-Vila, P., & Perry, S. (2012). Participation in group defence: Proximate factors affecting male behaviour in wild white-faced capuchins. *Anim. Behav. 83*(3), 621–628. https://doi.org/10.1016/j.anbehav.2011.12.001

Mirville, M. O., Ridley, A. R., Samedi, J. P. M., Vecellio, V., Ndagijimana, F., Stoinski, T. S., & Grueter, C. C. (2020). Intragroup Behavioral Changes Following Intergroup Conflict in Mountain Gorillas (Gorilla beringei beringei). *Int. J.Primatol*, *41*(2), 382–400. https://doi.org/10.1007/s10764-020-00130-1

Moher, D., Liberati, A., Tetzlaff, J., & Altman, D. G. (2009). Preferred reporting items for systematic reviews and meta-analyses: The PRISMA statement. *BMJ* 339, b2535, Issue 7716, pp. 332–336. https://doi.org/10.1136/bmj.b2535

Mosser, A., & Packer, C. (2009). Group territoriality and the benefits of sociality in the African lion, Panthera leo. *Anim. Behav. 78*(2), 359–370. https://doi.org/10.1016/j.anbehav.2009.04.024

Packer, C., Scheel, D., & Pusey, A. E. (1990). Why lions form groups: food is not enough. *Am. Nat. 136*(1), 1–19. https://doi.org/10.1086/285079

Putland, D. A., & Goldizen, A. W. (1998). Territorial behaviour in the Tasmanian native hen: group and individual performance. *Anim. Behav. 56*, 1455-1463.

Radford, A. N., & Du Plessis, M. A. (2004). Territorial vocal rallying in the green woodhoopoe: Factors affecting contest length and outcome. *Anim. Behav. 68*(4), 803–810. https://doi.org/10.1016/j.anbehav.2004.01.010

Robinson, J. G. (1988). Group size in wedge-capped capuchin monkeys Cebus olivaceus and the reproductive success of males and females. In *Behav. Ecol. Sociobiol. 23,* 187-197.

Roth, A. M., & Cords, M. (2016). Effects of group size and contest location on the outcome and intensity of intergroup contests in wild blue monkeys. *Anim. Behav*, *113* (June), 49–58. https://doi.org/10.1016/j.anbehav.2015.11.011

Scarry, C. J. (2013). Between-group contest competition among tufted capuchin monkeys, Sapajus nigritus, and the role of male resource defence. *Anim. Behav. 85* (5), 931–939. https://doi.org/10.1016/j.anbehav.2013.02.013

Sillero-Zubiri, C., & Macdonald, D. W. (1998). Scent-marking and territorial behaviour of Ethiopian wolves Canis simensis. *Journal of Zoology*, *245*(3), 351–361. https://doi.org/10.1017/S0952836998007134

Teichroeb, J. A., & Sicotte, P. (2018). Cascading competition: the seasonal strength of scramble influences between-group contest in a folivorous primate. *Behav. Ecol. Sociobiol. 72:6*(1). https://doi.org/10.1007/s00265-017-2418-x

Vessey, S. H. (1968). Interactions between free-ranging groups of rhesus monkeys. *Folia Primat.*, *8*, 228–239.

Zhang, P., & Watanabe, K. (2012). Variation in intergroup encounters among two provisioned free-ranging populations of Japanese macaques Macaca fuscata. Current Zoology, *58*(4), 517–524.
